# Individual and combined effects of cannabis and tobacco on drug reward processing in non-dependent users

**DOI:** 10.1007/s00213-017-4698-2

**Published:** 2017-07-22

**Authors:** Chandni Hindocha, Will Lawn, Tom P. Freeman, H. Valerie Curran

**Affiliations:** 1 Clinical Psychopharmacology Unit, University College London, Gower St, London, WC1E 6BT UK; 2 National Addiction Centre, Institute of Psychiatry, Psychology and Neuroscience, King’s College London, London, SE5 8BB UK

**Keywords:** Cannabis, Tobacco, Co-administration, Behavioural economics, Craving, Reward, Liking

## Abstract

**Rationale:**

Cannabis and tobacco are often smoked simultaneously in joints, and this practice may increase the risks of developing tobacco and/or cannabis use disorders. Currently, there is no human experimental research on how these drugs interact on addiction-related measures.

**Objectives:**

This study aimed to investigate how cannabis and tobacco, each alone and combined together in joints, affected individuals’ demand for cannabis puffs and cigarettes, explicit liking of drug and non-drug-related stimuli and craving.

**Method:**

A double-blind, 2 (active cannabis, placebo cannabis) × 2 (active tobacco, placebo tobacco) crossover design was used with 24 non-dependent cannabis and tobacco smokers. They completed a pleasantness rating task (PRT), a marijuana purchase task (MPT) and a cigarette purchase task (CPT) alongside measures of craving pre- and post-drug administration.

**Results:**

Relative to placebo cannabis, active cannabis reduced liking of cannabis-associated stimuli and increased response time to all stimuli except cigarette-related stimuli. Relative to placebo cannabis, active cannabis decreased demand for cannabis puffs (trends for breakpoint and elasticity) and cigarettes (breakpoint, *P*_max_, *O*_max_) on several characteristics of the purchase tasks. We found no evidence that active tobacco, both alone or combined with cannabis, had an effect on liking, demand or craving.

**Conclusions:**

Acutely, cannabis reduced liking of cannabis-related stimuli and demand for cannabis itself. Acute cannabis also reduced demand for cigarettes on the CPT. Acute tobacco administration did not affect demand or pleasantness ratings for cigarettes themselves or cannabis. In non-dependent cannabis and tobacco co-users, tobacco did not influence the rewarding effects of cannabis.

**Electronic supplementary material:**

The online version of this article (doi:10.1007/s00213-017-4698-2) contains supplementary material, which is available to authorized users.

## Introduction

Cannabis and tobacco are often smoked at the same time in the same preparation (e.g. in a ‘joint’ or ‘spliff’), and this is referred to as co-administration. Prevalence of simultaneous use of cannabis with tobacco amongst cannabis users is very high in the UK (77.2%) and across Europe (81.4–90.9%) as well as Australasia (20.7–51.6%) with the most prevalent route of administration being joints with tobacco (Hindocha et al. 2016). Although both drugs have reinforcing effects (Justinova et al. 2008; Shoaib et al. 1997), the cumulative probability of developing dependence across one’s lifetime is 67.5% for tobacco users and 8.9% for cannabis users, suggesting that tobacco is more addictive than cannabis (Lopez-Quintero et al. 2011). In a recent study of young adults in the UK, it was found that cigarette smoking increased the addictive potential of cannabis as it mediated the relationship between the frequency of cannabis use and dependence on the drug itself (Hindocha et al. 2015b). However, none of these studies adequately encapsulates the impact of co-administration with tobacco, which can acutely influence the subjective and cognitive effects of cannabis (Hindocha et al. 2017a; Schuster et al. 2016). The observational data is further limited as it is extremely challenging to disassociate the effects of cannabis from tobacco and to further isolate the acute effects of the drugs from residual and chronic drug effects.

Delta-9-tetrahydrocannabinol (THC), the primary psychoactive component of cannabis, and nicotine are partial agonists at the cannabinoid receptor 1 (e.g. CB1R) and acetylcholine receptors (nAChr), respectively. Preclinical data suggests a functional and bidirectional relationship between the cannabinoid and cholinergic systems that may be mediated by structures involved in motivation (Cohen et al. 2002). For example, prior exposure to THC increases the addictive effects of nicotine (Panlilio et al. 2013). The CB1R is critical to the rewarding effects of nicotine, such that in CB1R knock-out mice, the rewarding effects of nicotine are null (Castañé et al. 2002). To our knowledge, there has been no research on how co-administered cannabis and tobacco may influence aspects of reward processing related to these drugs in humans.

Berridge and Robinson (2003) separated reward processing into distinct components including ‘wanting’ and ‘liking’, each with separate implicit and explicit sub-components. The demand for drugs, relative to money, can be measured by purchase tasks (MacKillop et al. 2008) which give a real-world indication of the value of drugs (Bickel et al. 2014) and most likely capture aspects of *explicit motivation*. Purchase tasks are behavioural economic measures that quantify the association between drug consumption and price (MacKillop and Murphy 2013; Murphy and MacKillop 2006). Performance on the cigarette purchase task (CPT) has been associated with nicotine dependence, daily smoking and objective measures such as carbon monoxide levels (Mackillop et al. 2016; MacKillop et al. 2008). The marijuana purchase task (MPT) has shown associations with age of onset, craving and frequency of cannabis use (Aston et al. 2015; Collins et al. 2014). Most recently, a state version of the MPT has shown sensitivity to experimentally induced craving (Metrik et al. 2016) whereby cannabis demand indices increased and participants became less sensitive to price after a cue-reactivity paradigm (Metrik et al. 2016). However, no study has yet to investigate how acute cannabis, both individually and in combination with tobacco, affects demand for cigarettes and cannabis.

*Explicit liking* of drug-associated stimuli also plays a role in the reinforcing value of a drug. It can be indexed by pleasantness rating task (PRT), and the response may be related to hedonic processes involved in drug abuse (Morgan et al. 2010). Users of cannabis with high levels of THC in it have been shown to have greater explicit liking of cannabis images (Morgan et al. 2010); however, acute cannabis intoxication also produces a satiety response and therefore reducing liking overall (Metrik et al. 2015). On the other hand, findings on the relationship between cigarette use and drug and non-drug reward processing have been mixed (Lawn et al. 2015; Mogg et al. 2003; Powell et al. 2002). It is possible that cannabis and tobacco together may affect the hedonic responses to both drug and non-drug rewards; however, this possibility has not been investigated. Indeed, both have been implicated in food responses where they have opposite effects, cannabis stimulates appetite, whilst nicotine appears to decrease appetite (Kirkham 2005; Picciotto 2003). We differentiate effects of cannabis, tobacco and their combination on the subjective liking associated with cannabis, cigarette and food stimuli.

The present study aimed to investigate how acute administration of cannabis and tobacco, both alone and combined, would influence demand (for cannabis puffs and cigarettes), explicit liking of pictorial stimuli (cannabis, cigarette, food and neutral) and craving for cannabis, cigarettes and food. In light of the legalisation and medicalisation of cannabis, in this study, we recruited recreational cannabis and tobacco using a group who was not dependent on either drug but may be vulnerable to the development of addiction. This population would not experience withdrawal symptoms and would not have interference from residual or chronic drug effects. This study is clinically relevant as it investigates acute effects of cannabis and tobacco, both individually and in a potentially at-risk group, thus allowing us to understand the mechanism by which these users may transition to harmful use/dependence.

We firstly hypothesised that administration of either drug alone would reduce demand, liking and craving for that substance because of satiety (e.g. administrating active cannabis would reduce demand/liking/craving for cannabis). Secondly, we hypothesised that administration of one drug would increase demand, craving and liking for the other substance because of the strong association between cannabis and tobacco in individuals who use both together (e.g. administering cannabis *without* active tobacco would increase demand/liking/craving for tobacco). Finally, we hypothesised that cannabis would increase craving/liking of food-related stimuli and predicted the opposite pattern for tobacco.

## Method

### Design and participants

A randomised, double-blind, placebo-controlled four-way crossover trial was used to investigate the acute effects of cannabis and tobacco, both alone and combined. Participants attended four sessions, separated by at least 1 week (>3 times the half-life of THC (Hindocha et al. 2015a)) in a randomised order determined by a Latin square. Previous data from this study has been published elsewhere focussing on memory and psychotomimetic effects (Hindocha et al. 2017a) and validation of self-reported dose per joint (Hindocha et al. 2017b). The estimated sample size of 24 was based on a previous four-way crossover study examining the interactive effects of THC and CBD (Hindocha et al. 2015a). This would achieve an effect size of *d* = 0.5 with 80% power at an alpha of 0.05 (G*power version 3.1.9.2) (Faul et al. 2007). Participants from the community were recruited through posters around London universities and on online notice boards. All participants provided written, informed consent. Ethical approval was given by the UCL Ethics Committee.

Inclusion criteria were (i) age 18–60 years; (ii) regular use (>once per month and <3 times a week) of cannabis mixed with tobacco in joints for the previous 6 months (Lawn et al. 2016); (iii) normal or corrected to normal vision; (iv) fluent English; (v) self-reported abstinence from tobacco, cannabis, alcohol and other drugs for at least 12 h prior to each test day; (vi) alveolar carbon monoxide (CO) ≤6 ppm to confirm no recent smoking on each test day (Cooper and Haney 2009); and (vii) self-reported ability to smoke one whole ‘standard’ joint which is considered a relatively high bar as common practise is to share joints in recreational users (Lawn et al. 2016).

Exclusion criteria were (i) scoring ≥3 on the Cannabis Severity of Dependence Scale (SDS; Gossop et al. 1995); (ii) seeking treatment for cannabis or tobacco use or currently using nicotine replacement therapy or other cessation pharmacotherapy; (iii) smoking ≥10 cigarettes a day or scoring ≥4 on the Fagerstrom Test of Nicotine Dependence (FTND (Heatherton et al. 1991)); (iv) first cigarette smoked within the first 3 h after waking (to ensure results were not simply due to reversal of withdrawal from tobacco (Jarvik et al. 2000)); (v) significant respiratory or physical disorder or a clinically diagnosed learning impairments; (vi) clinically diagnosed schizophrenia or psychosis (or a first-degree family member with either) or substance use disorder and (vii) use of illicit substance use other than cannabis more than once per week.

### Drug administration (Fig. 1/Table 1)

**Fig. 1:**
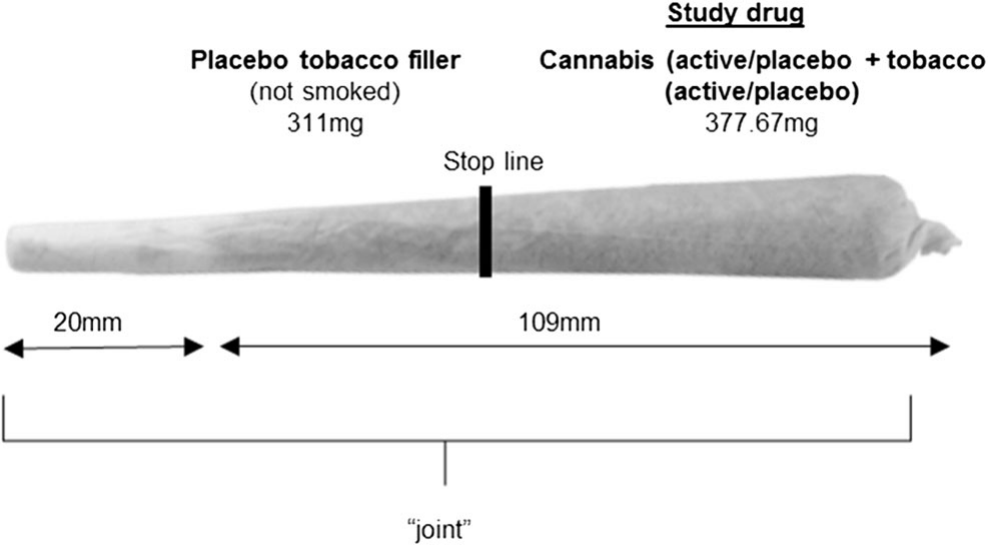
Drug administration was conducted using ‘joints’, the most common method of administering cannabis in the Europe and Australasia (Hindocha et al. 2016). ‘Study drug’ region contained a mixture of 66.67-mg cannabis (active or placebo) and 311-mg tobacco (active or placebo) dependent on condition (see Table 1). The ‘placebo tobacco filler’ region contained 311 mg of placebo tobacco at the *bottom* of the joint (nearest to the mouth) which was not smoked. This filler was added to improve compliance with the fixed inhalation procedure, as puff volume typically decreases towards the end of the joint, probably due to rising heat (van der Pol et al. 2014). The stop line is the point at which participants stopped smoking the joint, separating the two regions. It was marked 1 cm after the ‘study drug’ to ensure complete inhalation

We compared the effects of (a) active cannabis + active tobacco (CAN-TOB), (b) active cannabis + placebo tobacco (CAN), (c) placebo cannabis + active tobacco (TOB) and (d) placebo cannabis + placebo tobacco (no active drug) (PLACEBO). The dose of cannabis and tobacco specified in Table 1 was based on previous experimental studies reporting robust subjective, cardiovascular, psychotomimetic and memory-impairing effects (Lawn et al. 2016; Mokrysz et al. 2016) for cannabis and a reliable increase in peak plasma nicotine levels of >20 ng/ml for tobacco (Mendelson et al. 2005; Mendelson et al. 2003). This is also similar to a standard cannabis + tobacco joint (Hunault et al. 2009; van der Pol et al. 2014). Placebo cannabis is produced from active cannabis and contains less than 0.1% THC (but with the same terpene content, so it retains the look and smells of cannabis). Placebo tobacco was the same dose of very low nicotine (VLN; typically referred to as denicotinised) tobacco (Magic 0 (XXII Century Group Ltd)). The smoking procedure was standardised to control for dose titration and maximise absorption of THC (Ramaekers et al. 2006). The smoking procedure was paced. Participants were asked to inhale for 4 s, hold their breath for 8 s and then exhale and break for 30 s. This sequence was repeated until the joint was smoked up to a designated line (Fig. 1). Drug administered took place in a sheltered outdoor area. This protocol was timed and enforced by the experimenter.

**Table 1:** Cannabis and tobacco doses in the study drug and their matched placebos for the four drug conditions

Drug	Condition	Description
Cannabis	Active	66.67 mg Bedrobinol (16.1% THC and <1% CBD).
	Matched placebo	66.67 mg placebo (derived from Bedrocan; 0.07% THC)
Tobacco	Active	311 mg Marlboro Red (15.48-mg nicotine, 16-mg tar, 0.8-mg nicotine yield).
	Matched placebo	311 mg denicotinised tobacco (Magic 0, 0.04 mg/g nicotine)

Both active and placebo cannabis were sourced from Bedrocan® and are commercially available

### Assessments

The pleasantness rating task (PRT) tapped explicit liking and response time to cannabis-, tobacco-, food- and neutral-related cues. In this computer-based task, participants were presented with a fixation cross (500 ms) followed by four types of pictorial stimuli in a randomised order for 3 s. Participants were asked to rate the pleasantness of each image on a scale of −3 (very unpleasant) to +3 (very pleasant). Stimuli were matched on brightness and complexity and included 36 critical trials. Pictorial stimuli for cigarettes involved smoking-related scenes and were used previously by Mogg et al. (2005). Neutral stimuli were taken from the International Affective Picture System (IAPS) (Lang et al. 1999). Cannabis and food pictorial stimuli were expanded from a previous stimulus set (Morgan et al. 2010). The task design was modified from Metrik et al. (2015). Four versions were used and counterbalanced across drug design. The experiment was built and conducted using Psychopy (Peirce 2007; Peirce 2009).

The marijuana purchase task (MPT) (Aston et al. 2015; Collins et al. 2014) and cigarette purchase task (CPT) (MacKillop et al. 2008) assess cigarette/cannabis demand, i.e. the relationship between cigarette/cannabis consumption and cost (Aston et al. 2015; MacKillop et al. 2008). It is an analogue of a progressive ratio operant task as consumption is investigated under progressively increasing financial cost. It is an established and well-validated task (Aston et al. 2015; Chase et al. 2013; MacKillop et al. 2008; Secades-Villa et al. 2016). In this version, participants were asked how many cigarettes/cannabis puffs they would hypothetically buy in the next 3 h at increasing prices (Hitsman et al. 2008; Lawn et al. 2017). Specifically, they were asked, ‘How many cigarettes would you smoke if they were _____ each’ or ‘How many puffs of cannabis would you smoke if they were _____ each’. Prices included £0 (free), 1p, 2p, 5p, 10p, 15p, 20p, 30p, 40p, 50p, 75p, £1, £1.50 £2, £2.50, £3, £3.50, £4, £5, £7.50, £10, £15 and £20 and were presented in that order for both the CPT and MPT. Five indices of cigarette/cannabis demand were generated: breakpoint (cost suppressing consumption to zero), intensity (amount of drug consumed at zero cost), *O*_max_ (peak expenditure), *P*_max_ (price at maximum expenditure) and elasticity (the slope of the demand curve). Importantly, adjustments were made for UK participants for the MPT, including replacing ‘marijuana’ with ‘cannabis’ and ‘hits’ with ‘puffs’. Full instructions for the CPT and the MPT are in Online Resource 1.

### Craving

This was assessed ‘right now’ at all five time points with three single-item visual analogue scales (VAS) for cannabis, tobacco and food. Each item began with ‘I am craving…’ with anchors ‘not at all’ and ‘extremely’.

### Subjective effects

This was assessed ‘right now’ at all five time points with two single-item VAS for euphoric and stimulated. Anchors were ‘not at all’ and ‘extremely’.

### Procedure

Participants attended a baseline session followed by four experimental sessions over a 4–6-week period. Eligibility was assessed by telephone screening and during the baseline session. Each experimental session began with pre-drug VAS for craving and subjective effects. After drug administration, participants completed further VAS for craving and subjective effects at four time points over the next hour as well as the CPT, MPT and PRT (see Schedule of Assessments in Online Resource 1). Other tasks that are not reported here took place in the intervening time (see Hindocha et al. 2017a). They were reimbursed £60 for their time on the last test day and debriefed fully.

### Statistical analysis

All data were analysed using IBM Statistical Package for Social Sciences (IBM SPSS version 23) and GraphPad Prism 7 for Windows (GraphPad Software, La Jolla California USA, www.graphpad.com). For the PRT, outliers >2.5 SD from the sample mean were replaced with a score falling within 2.5 SD of the mean following Das et al. (2015). Normality was explored using visual inspection of diagnostic plots. When sphericity was violated, the Greenhouse-Geisser correction was used and corrected degrees of freedom are reported. For the PRT, we conducted a 2 (cannabis, placebo) × 2 (tobacco, placebo) × 4 (picture type) repeated measures ANOVA on both valence and response time measures.

Data from the purchase tasks was examined for outliers using standard scores (Z), with a criterion of *Z* = 3.29 to retain maximum data (Tabachnick and Fidell 2000). Of the data, 0.02% were outliers (Tabachnick and Fidell 2000). The outliers were determined to be legitimate high-magnitude values and were re-coded as one unit higher than the next lowest non-outlying value as per Aston et al. (2015) (Tabachnick and Fidell 2000). Zero data (i.e. when participants responded that they would not buy purchase any cannabis or cigarettes for 0p, i.e. free) was calculated as 41% (39/96 data points) for the CPT and 7% (7/96 data points) for the MPT, and this was due to floor effects post-drug administration. Annual income was considered as a potential covariate, but as it did not correlate with demand indices under any drug (*p* > 0.09), it was not included (MacKillop et al. 2012). Each demand characteristic was analysed using mixed-effects models, which accounts for missing data whilst behaving like a repeated measures ANOVA. Cannabis (active, placebo) and tobacco (active, placebo) were entered as fixed effects, and the intercept was allowed to vary randomly. Breakpoint, intensity, *O*_max_ and *P*_max_ were directly observed from the data. Price elasticity was generated using a modification of the non-linear exponential demand curve model (Koffarnus et al. 2015): *Q* = *Q*_0_ × 10^*k*(^ *e* ^−*αP*−1)^, where *Q* = quantity consumed, *Q*_0_ = derived intensity, *k* = a constant across individuals that denotes the range of the dependent variable (cannabis puffs or cigarettes) in logarithmic units, *P* = price and *α* = elasticity or the rate constant determining the rate of decline in log consumption based on increases in price (i.e. essential value). *k* was fixed to log(80) = 1.9 for the MPT and log(9) = 0.9 for the CPT. *Q*_0_ was fitted as consumption at 0 pence (free), i.e. intensity. This is a modification of the Hursh and Silberberg (2008) exponential demand equation and avoids poor model fit because of exclusion of zeros in the equation (Yu et al. 2014).

VAS scores had an additional task-specific factor of time, which was investigated using Helmert contrasts for time (1 (pre-drug) vs 2, 3, 4, 5 (post-drug)).

## Results

### Demographic and drug use history

Participants were 24 (50% female) recreational cannabis and tobacco co-users. All participants completed all assessments. Demographics and drug use variables can be found in Table 2.

**Table 2:** Demographics and drug history of participants

	*N* = 24 (mean, SD)
% Female	50%
Age (years)	24.46 ± 3.96
SDS	0.67 ± 0.92 (range: 0–3)
FTND	0.33 ± 0.64 (range: 0–2)
Annual income (£)	14,238.33 ± 10,324.83
Cannabis + tobacco	
Age of first use (years)	16.16 ± 3.94
Last used (days)	7.92 ± 9.64
Years used (years)	6.79 ± 3.94
Days per month	7.75 ± 4.43
Time to smoke 3.5 g (days)	36.58 ± 34.47
Lifetime exposures (days)	627 ± 936
Exposures in the last 90 days (days)	19.58 ± 11.27
Tobacco alone	
Age of first use (years)	15.71 ± 1.94
Last used (days)	96.13 ± 313.26
Years used (years)	6.76 ± 4.58
Days per month	11.04 ± 12.68
Cigarettes per day	2.29 ± 2.74
Lifetime exposures (days)	2834 ± 7202
Exposures in the last 90 days (days)	29.75 ± 33.56
Cannabis alone	
Age of first use (years)	16.32 ± 5.41
Last used (days)	466.86 ± 866.37
Years used (years)	3.31 ± 4.16
Days per month	0.82 ± 2.09
Lifetime exposures (days)	49.18 ± 97.60
Exposures in the last 90 days (days)	3.55 ± 6.39

### Pleasantness rating task (Fig. 2)

#### Valence (Fig. 2a)

**Fig. 2:**
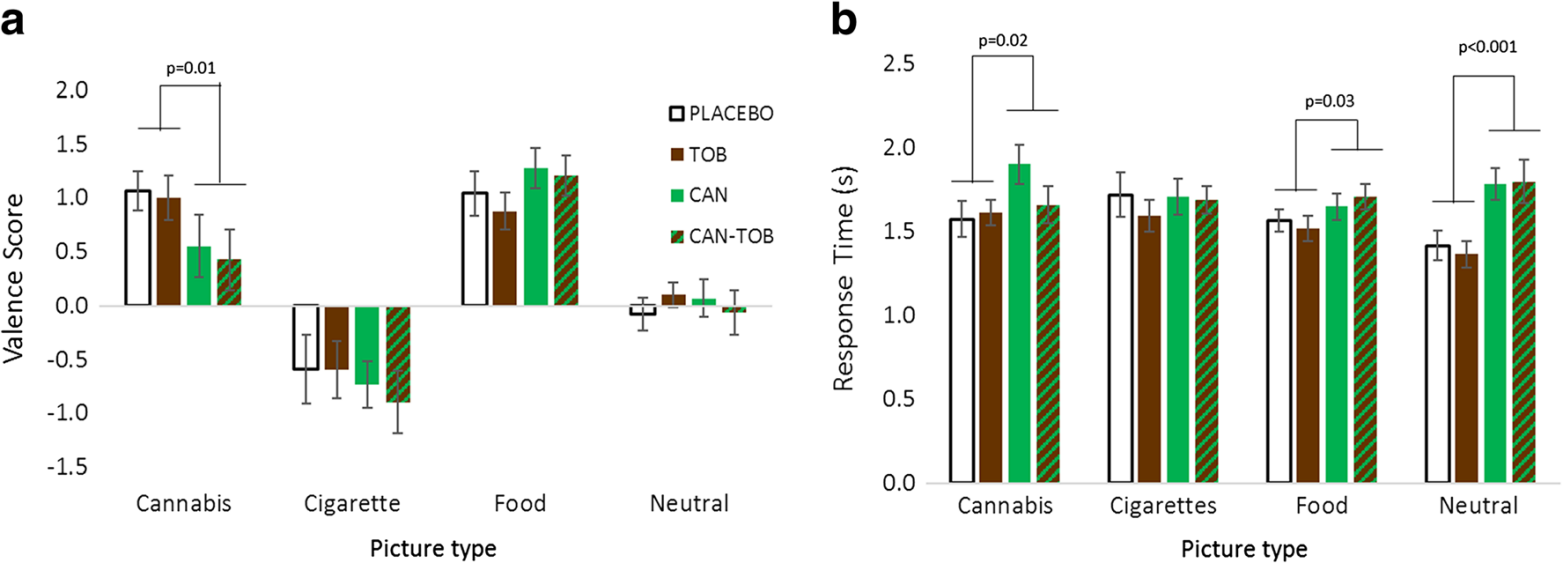
**a** Valence score dependent on drug condition for each picture type. **b** reaction time dependent on drug for each picture type (*error bars* show ±SEM)

There was a cannabis × picture-type interaction (*F*_3,69_ = 5.35, *p* = 0.002, *η*_p_^2^ = 0.19) whereby cannabis stimuli were rated as less pleasant under active than placebo cannabis (*p* = 0.01; Fig. 2a). Food stimuli were rated as more pleasant under cannabis than placebo at a trend level (*p =* 0.053). There was a main effect of picture type (*F*_3,69_ = 20.68, *p* < 0.001, *η*_p_^2^ = 0.47). Tobacco was rated as unpleasant across all drug conditions and neutral stimuli as around zero valence.

#### Response time (Fig. 2b)

We found a cannabis × picture-type interaction (*F*_3,69_ = 6.60, *p* = 0.001, *η*_p_^2^ = 0.223) and a main effect of cannabis (*F*_1,23_ = 20.33, *p* < 0.001, *η*_p_^2^ = 0.47). The interaction suggests that cannabis acutely slowed response time across all stimuli apart from cigarette stimuli.

### Purchase tasks

Means (+SEM) for the demand indices derived from the MPT and CPT for each drug condition can be found in Table 3.

**Table 3:** Means (SEM) for the demand indices derived from the cigarette purchase task (CPT) and the cannabis purchase task (MPT) for each drug condition

	Drug condition			
	CAN-TOB	CAN	TOB	PLACEBO
CPT				
Breakpoint	81.67 (17.79)	97.19 (19.94)	134.64 (26.24)	139.64 (19.75)
Intensity	4.50 (0.96)	4.00 (0.84)	3.86 (0.73)	3.75 (0.67)
*O*_max_	107.08 (24.78)	122.50 (28.96)	193.57 (53.07)	149.28 (26.49)
*P*_max_	50 (12.08)	56.56 (13.45)	87.14 (20.18)	76.79 (13.47)
Elasticity	1.65 (0.86)	2.52 (0.78)	1.84 (0.83)	1.03 (0.83)
MPT				
Breakpoint	164.75 (48.99)	145.29 (33.23)	254.63 (84.25)	214.00 (58.40)
Intensity	16.00 (3.52)	17.14 (3.40)	15.63 (2.05)	15.67 (2.29)
*O*_max_	556.00 (143.53)	652.95 (183.86)	621.71 (123.21)	721.87 (162.47)
*P*_max_	65.55 (3.20)	92.19 (24.21)	81.50 (17.88)	122.50 (41.67)
Elasticity	0.27 (0.19)	0.61 (0.18)	0.11 (0.17)	0.17 (0.17)

#### Marijuana purchase task

There was a trend towards a main effect of cannabis on breakpoint (*F*_1,62_ = 3.89, *p =* 0.053) where active cannabis reduced the first price at which consumption was zero, in comparison to placebo cannabis. There was a trend towards a main effect of cannabis on elasticity (*F*_1,668_ = 2.94, *p* = 0.09), where cannabis increased sensitivity to cost, in comparison to placebo. There were no other main effects or interactions with tobacco for the other demand indices (MPT intensity, *O*_max_ or *P*_max_).

#### Cigarette purchase task (Fig. 3)

**Fig. 3:**
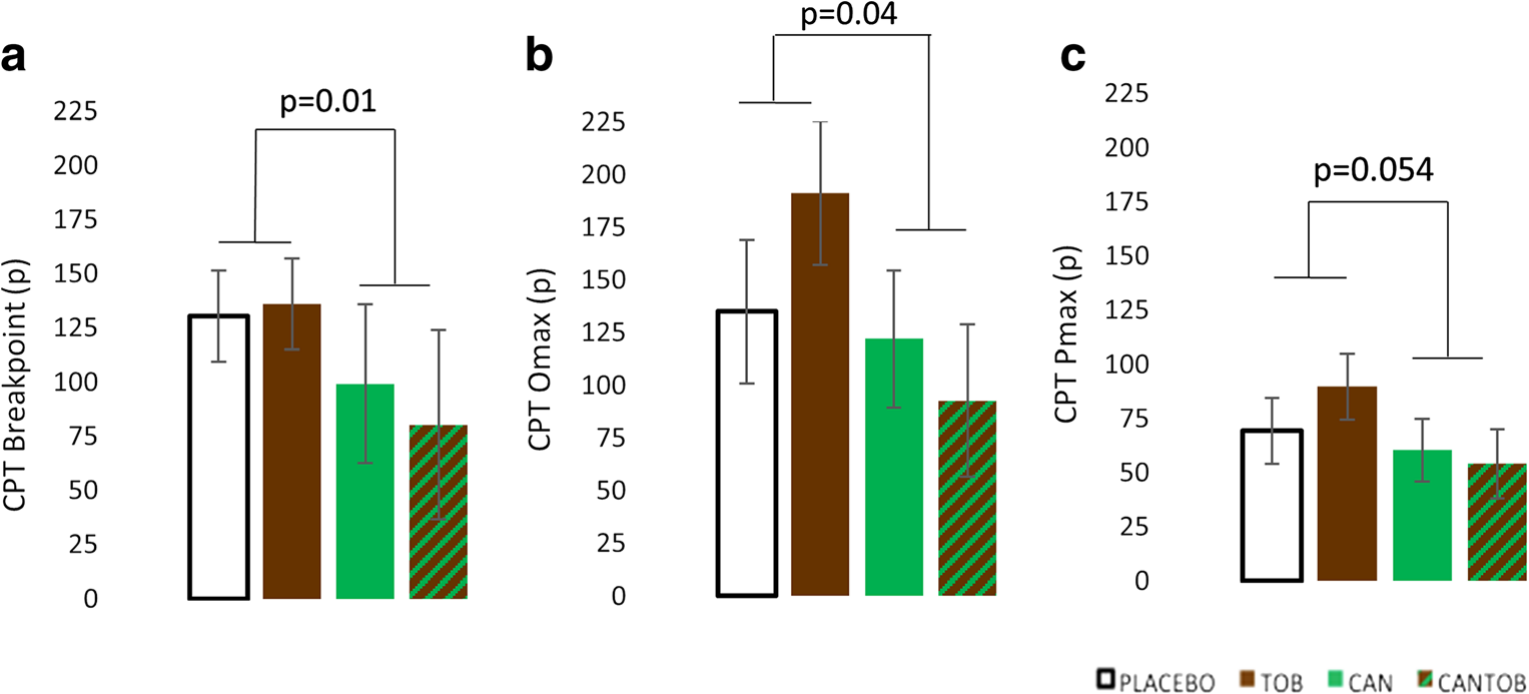
CPT indices. There were main effects for cannabis on **a** breakpoint, **b** *O*_max_ and **c** *P*_max_ (trend main effect) (*error bars* show ±SEM)

There was a main effect of cannabis on breakpoint (*F*_1,37.37_ = 7.00, *p* = 0.01) where cannabis decreased the breakpoint in comparison to placebo (Fig. 3a). There was a main effect of cannabis for the *O*_max_ (*F*_1,38.94_ = 4.37, *p* = 0.04) (Fig. 3b) where cannabis reduced the maximum expenditure. There was a trend for a main effect of cannabis for the *P*_max_ (*F*_1,35.54_ = 3.97, *p =* 0.054) where cannabis also reduced the price of the maximum expenditure for cigarettes (Fig. 3c). For all the above demand indices, there was no interaction with tobacco. There were no main effects or interactions for the other CPT demand indices (i.e. intensity and elasticity).

### Craving (Fig. 4)

#### Crave food (Fig. 4a)

There was a trend towards a main effect of tobacco (*F*_1,23_ = 4.11, *p* = 0.054, *η*_p_^2^ = 0.15); across all time points, tobacco reduced craving for food in comparison to placebo. There was also a main effect of time (*F*_1,23_ = 38.58, *p* < 0.001, *η*_p_^2^ = 0.63) so participants craved food more as the test session progressed.

**Fig. 4:**
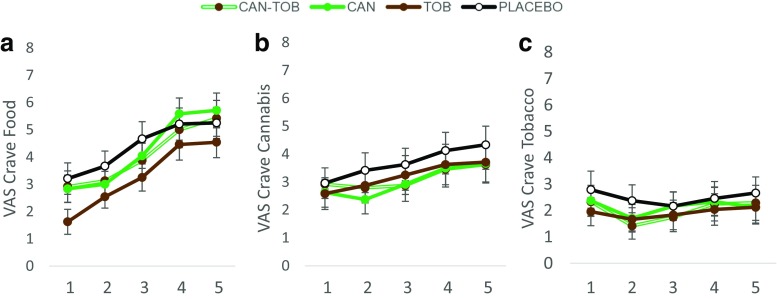
Subjective ratings of craving for **a** food, **b** cannabis and **c** tobacco, averaged across all participants for all time points before (T1) and after (T2–T5) each drug administration. *Error bars* represent ±SEM

#### Crave cannabis (Fig. 4b)

There was a main effect of time (*F*(1, 23) = 5.80, *p* = 0.025 *η*_p_^2^ = 0.20) but no other main effects or interactions.

#### Crave tobacco (Fig. 4c)

There were no main effects or interactions for VAS crave tobacco.

### Subjective effects

#### Euphoric (Fig. 5a)

There was a cannabis × time interaction (*F*_1,23_ = 18.13, *p* < 0.001, *η*_p_^2^ = 0.44) which revealed a significant increase between cannabis and placebo from pre- to post-drug. Pre-drug, there was no difference between active and placebo cannabis (*p* = 0.178); however, active cannabis increased ‘euphoric’ ratings at all time points post-drug (all *p*s ≤ 0.004). There were also main effects of cannabis (*F*_1,23_ = 10.79, *p* = 0.003, *η*_p_^2^ = 0.32) and time (*F*_1,23_ = 12.87 *p* = 0.002, *η*_p_^2^ = 0.36). There were no main effects or interactions with tobacco.

**Fig. 5:**
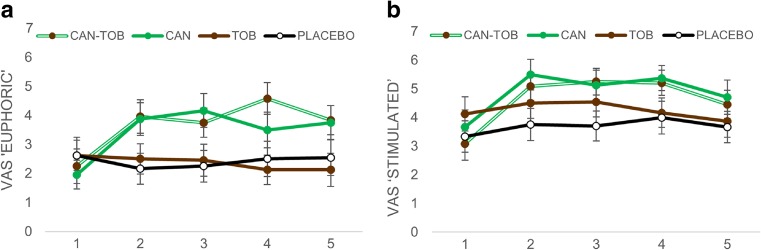
Subjective ratings of VAS **a** ‘euphoric’ and **b** ‘stimulated’ averaged across all participants for all time points before (T1) and after (T2–T5) each drug administration. *Error bars* represent ±SEM

#### Stimulated (Fig. 5b)

There was a cannabis × time interaction (*F*_1,23_ = 6.84, *p* = 0.016, *η*_p_^2^ = 0.23) which revealed a significant increase between cannabis and placebo from pre- to post-drug. Pre-drug, there was no difference between active and placebo cannabis (*p* = 0.437); however, active cannabis increased ‘stimulated’ ratings at all time points post-drug (all *p*s < 0.05). There were also main effects of cannabis (*F*_1,23_ = 5.82, *p* = 0.024, *η*_p_^2^ = 0.20) and time (*F*_1,23_ = 11.52, *p* = 0.002, *η*_p_^2^ = 0.33). There were no main effects or interactions with tobacco.

## Discussion

To the authors’ knowledge, this is the first study to examine both the individual and combined effects of cannabis and tobacco on drug reward processing. We found that, compared with placebo, acute cannabis reduced liking of cannabis- (but not cigarette-) associated stimuli and increased response time to rate all picture types apart from cigarettes. Acute cannabis administration reduced, although not significantly, the first point where demand was zero (breakpoint) for both cannabis puffs and cigarettes, in comparison to placebo. We saw reduced maximum expenditure (*P*_max_) and *O*_max_ for cigarettes; however, this was not significant for *P*_max_ and therefore should be interpreted with caution until it can be replicated. Overall, this suggests that participants under the influence of cannabis became *more* sensitive to price increases and therefore less likely to buy cigarettes or cannabis at higher prices. Smoked tobacco either alone or combined with cannabis affects demand indices for cannabis or cigarettes. Taken together, acute administration of cannabis reduced, to a degree, demand for both cannabis and cigarettes. Finally, active cannabis increased ratings of both euphoric and stimulated, but tobacco had no effect on these ratings. From a public health and clinical perspective, health-focussed campaigns should emphasise that adding tobacco to cannabis does not modify the reward processing of cannabis, and thus, users should be dissuaded from mixing cannabis with tobacco. The present results could be a product of cross-satiety between the two drugs because this population use cannabis and tobacco together like many in Europe such that consuming cannabis also reduces demand for tobacco (Hindocha et al. 2016).

Moreover, we found that there was a trend (i.e. not significant) towards acute cannabis administration increasing elasticity for cannabis puffs, indicating that participants were slightly more sensitive to the price of cannabis. This is in line with a recent study by Metrik et al. (2016) where experimentally induced craving *reduced* elasticity making participants *less* sensitive to price and suggesting continued purchasing despite price increases (Metrik et al. 2016). The present results and that of Metrik et al. (2016) are in opposite directions and together show that the state MPT is sensitive to both satiety via acute administration and cue-elicited craving. There were no main effects or interactions with tobacco, suggesting that consumption of tobacco does not alter demand for cannabis in this specific context. Future research should investigate under conditions of cue reactivity, for both cannabis and tobacco, if cross-cue elicited craving occurs and if there would be a knock-on effect on demand. It should be noted that a possible reason why there was a minimal effect on demand for cigarettes is because participants were non-dependent cigarette smokers and little research has been carried out on demand, as measured by purchase tasks, in non-dependent smokers (‘chippers’) (Shiffman 1989). In this study, we investigated a non-dependent population, which is an important line of investigation as non-dependent, but regular users are vulnerable to the development of addiction and the acute effects of the drugs are not affected by residual drug use or withdrawal.

In the present study, we found that active cannabis reduced liking of cannabis stimuli consistent with research suggesting that cannabis users find cannabis-related stimuli more pleasant under placebo than active cannabis (Metrik et al. 2015). Cannabis stimuli were always rated as pleasant (regardless of drug condition), but after smoking active cannabis, the ratings reduced indicative of satiety. Moreover, we found some evidence that cannabis and tobacco had opposite effects on food responses; i.e. cannabis tended to increase liking of food stimuli, consistent with classic cannabis-induced ‘munchies’, and tobacco decreased craving for food, as hypothesised. Interestingly, we did not see an equivalent effect of food craving, and it is logical that these two would increase concurrently. This may be because the pictorial stimuli of the task were more hunger-inducing than a single-item question. Indeed, food craving did increase steadily over time, but no drug effect emerged. Under all conditions, cigarette stimuli were rated as more unpleasant than all other stimuli, and cannabis slowed response times to all stimuli except cigarettes. This may be because participants had little to no dependence on cigarettes; however, it may also be due to the negative connotations and stigma associated with tobacco. Young cannabis users often do not consider themselves tobacco smokers even though it facilitates cannabis use and is significantly exposing them to tobacco and its by-products (Bélanger et al. 2011). Perhaps because of their strong negative valence, response times to tobacco stimuli were not modified by acute cannabis. Moreover, it should be noted that neutral stimuli were rated with zero valence, showing that they were indeed rated as neutral. Future research will be required to investigate if there is a different pattern of results in dependent users of cannabis and tobacco, who may be more sensitive to tobacco cues, and this may vary by acute drug intoxication. Future research might also investigate self-administration of individual and combined cannabis and tobacco in humans which would give direct demonstration of the abuse potential of the drugs combined relative to their components; however, that was not the aim of the present study.

## Strengths and limitations

This study has several strengths including its sample size informed by a power calculation, an ecologically valid method of drug administration and factorial investigation of cannabis and tobacco in a double-blind placebo-controlled design. Moreover, we attempted to control for both drugs by asking participants to abstain for at least 12 h and we were able to confirm this for tobacco with a CO level of <6 on each test day. We also attempted to control for food intake by asking participants not to eat for at least 2 h before each testing day. However, we were not able to verify (beyond the self-reported SDS) that participants did not have a cannabis use disorder although the mean SDS score was low (0.67 ± 0.92). The lack of effects detected for tobacco are unlikely to be due to an insufficient dose, as we also found that cannabis and tobacco had significant and opposite effects of memory (Hindocha et al. 2017a). Indeed, adding tobacco to cannabis attenuated the negative effect of cannabis on delayed recall in a verbal memory task. Moreover, the lack of effect on reward-related measures is unlikely to be due to a negative response to the drug because ratings of euphoric and stimulated increased significantly, but there was no difference between mixed cannabis and tobacco in comparison to cannabis alone. Moreover, we found that cannabis and tobacco had independent effects on increasing heart rate and interacting effects on increasing diastolic blood pressure (Hindocha et al., 2017a). The doses and route of administration of cannabis and tobacco were designed to be comparable to real-life use, and the inclusion criteria of smoking one ‘whole’ joint are considered a high bar as recreational users mostly share joints. Finally, when participants experienced satiety, they stated that they would not buy any hypothetical cannabis puffs, which led to 41% of zero data (i.e. when participant would not purchase puffs for zero pence; floor effects). Though we chose a method of analysis that would allow us to control for this, this is a substantial proportion of the data, and therefore, these results need to be interpreted cautiously. It indicates the need for more suitable state instruments, which do not result in floor effects because of satiety. Finally, future studies should include comparative purchase tasks for food and validate a purchase task for cannabis-tobacco joints.

## Conclusions

In view of current changes in the medicalisation and legalisation of cannabis, research regarding cannabis and tobacco on addiction-related outcomes is essential. This study aimed to investigate how cannabis and tobacco, alone and combined, would affect validated addiction-related outcomes such as drug demand, explicit liking of associated stimuli and craving, in recreational cannabis and tobacco joint smokers. This study further helps us understand the mechanism by which recreational users may transition to harmful or dependent patterns of use. We found that, acutely, cannabis reduced liking of cannabis stimuli and reduced demand for both cannabis puffs and cigarettes in the purchase task. In this population, tobacco did not influence the rewarding effects of cannabis. Therefore, health campaigns should try to dissuade users from adding tobacco to cannabis, as it does not make cannabis more rewarding.

## Electronic supplementary material

ESM 1 (DOCX 39 kb).
