# Supplementary material for: Individual and combined effects of cannabis and tobacco on drug reward processing in non-dependent users
Source: Psychopharmacology (Berl). 2017 Jul 22;234(21):3153–63. doi: 10.1007/s00213-017-4698-2 (PMC5660839; doi:10.1007/s00213-017-4698-2)
Supplement: Supplementary file 1 — (DOCX 39 kb). [file 213_2017_4698_MOESM1_ESM.docx]

**ONLINE RESOURCE 1**

**Individual and combined effects of cannabis and tobacco on drug reward processing in non-dependent users**

Chandni Hindocha^1^, Will Lawn^1^, Tom P Freeman^1^ , H. Valerie Curran^1^

^1^Clincial Psychopharmacology Unit, University College London, Gower St, London, UK

**Cigarette Purchase task (CPT) and Marijuana Purchase Task (MPT)**

*Instructions for the CPT were the following:*

*The following questions ask how many cigarettes you would purchase at various prices, if they were offered to you RIGHT NOW for over the next THREE HOURS.  The following questions ask how many cigarettes you would consume if they cost various amounts of money, assuming that the available cigarettes are your favorite brand and that you have NO ACCESS to any other cigarettes/nicotine now or after this session. The available cigarettes are your favorite brand. Answer each question individually, i.e. the number you would buy for price X should not affect the number you would buy for price Y. You cannot save or stockpile cigarettes for a later date. There are no limits on how many cigarettes you can purchase*

*Instructions for the MPT were the following:*

*The following questions ask how many PUFFS of cannabis you would purchase at various prices, if they were offered to you RIGHT NOW for over the next THREE HOURS. Assume that you have to smoke all the cannabis that you purchase, and that you cannot get any more cannabis now or after this session. The cannabis is of AVERAGE quality and strength. The joint DOES NOT have any tobacco in it. Answer each question individually, i.e. the number you would buy for price X should not affect the number you would buy for price Y. How many puffs of marijuana would you take RIGHT NOW at the following prices? There are 10 puffs of cannabis in a joint. There is no limit on puffs or joints.*

*
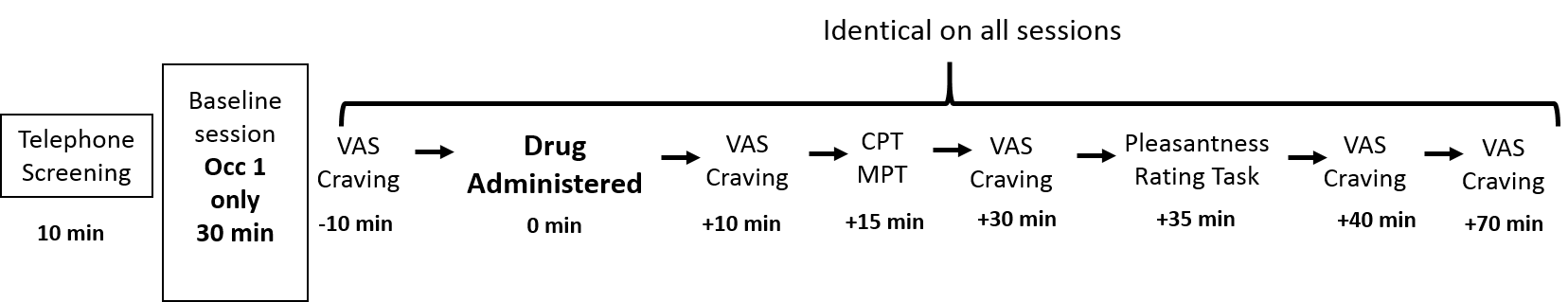
*

*Online Resource Figure 1. Schedule of Assessments. Other tasks that are not reported here were undertaken at the intervening time points. Post-drug timings are from the beginning of smoking onset. Other tasks that are not reported here took place in the intervening time. Abbreviations – VAS: visual analogue scale; CPT: cigarette purchase task; MPT: marijuana purchase task.*
